# *Plasmodium *infection alters *Anopheles gambiae *detoxification gene expression

**DOI:** 10.1186/1471-2164-11-312

**Published:** 2010-05-19

**Authors:** Rute C Félix, Pie Müller, Vera Ribeiro, Hilary Ranson, Henrique Silveira

**Affiliations:** 1 Centro de Malária e Outras Doenças Tropicais, UEI Malária, Instituto de Higiene e Medicina Tropical, Universidade Nova de Lisboa, Rua da Junqueira, 96, 1349-008 Lisbon, Portugal; 2 Vector Group, Liverpool School of Tropical Medicine, Liverpool, L3 5QA, UK; 3 Vector Control Unit, Medical Department, Swiss Tropical and Public Health Institute, CH-4002 Basel, Switzerland; 4 University of Basel, CH-4003 Basel, Switzerland; 5 Centro de Biomedicina Molecular e Estrutural (CBME), Instituto de Biotecnologia e Bioengenharia (IBB-LA), Universidade do Algarve, Faro, Portugal

## Abstract

**Background:**

*Anopheles gambiae *has been shown to change its global gene expression patterns upon *Plasmodium *infection. While many alterations are directly related to the mosquito's innate immune response, parasite invasion is also expected to generate toxic by-products such as free radicals. The current study aimed at identifying which loci coding for detoxification enzymes are differentially expressed as a function of *Plasmodium berghei *infection in midgut and fat body tissues.

**Results:**

Using a custom-made DNA microarray, transcript levels of 254 loci primarily belonging to three major detoxification enzyme families (glutathione S-transferases, cytochrome P450 monooxygenases and esterases) were compared in infected and uninfected mosquitoes both during ookinete invasion and the release of sporozoites into the hemocoel. The greatest changes in gene expression were observed in the midgut in response to ookinete invasion. Interestingly, many detoxification genes including a large number of P450s were down-regulated at this stage. In the fat body, while less dramatic, gene expression alterations were also observed and occurred during the ookinete invasion and during the release of sporozoites into the hemocoel. While most gene expression changes were tissue-related, *CYP6M2*, a CYP previously associated with insecticide resistance, was over-expressed both in the midgut and fat body during ookinete invasion.

**Conclusions:**

Most toxicity-related reactions occur in the midgut shortly after the ingestion of an infected blood meal. Strong up-regulation of *CYP6M2 *in the midgut and the fat body as well as its previous association with insecticide resistance shows its broad role in metabolic detoxification.

## Background

The mosquito *Anopheles gambiae *is the main malaria vector in sub-Saharan Africa. Resistance to anti-malaria drugs and insecticides together with the lack of vaccines highlight the need for novel strategies in malaria control. Such a strategy could be the interruption of the transmission cycle within the mosquito.

The mosquito becomes infected with the malaria parasite by taking a blood meal. The blood meal itself brings metabolic changes and induces a state of oxidative stress [1,2]. This is further increased by the presence of *Plasmodium *parasites in the blood meal [3]. During mosquito response to infection, active nitrogen and oxygen radicals are produced to contain *Plasmodium *infection [1,3]. These products may represent potential oxidative stress that can be ameliorated or eliminated by detoxification enzymes. For example several glutathione S-transferases (GSTs) have peroxidase activity and some can also metabolise reactive α,β-aldehydes [4]. GST expression can also be induced by reactive oxygen species (ROS) [5,6]. While GSTs help to eliminate ROS, cytochrome P450 monooxygenases (CYP) may actually contribute towards its generation [7].

Although transcription alteration of detoxification genes in response to bacteria and *Plasmodium *[8-10] has been described, the nature of this response hasn't been fully discussed. In this study we describe the impact of *P. berghei *infection at two time points (1 day and 11 days post infection) on the expression of detoxification genes in the midgut and fat body. We identified several genes, previously implicated in the detoxification of xenobiotics, which are differentially expressed in relation to parasite infection in the midgut and fat body. The possible role of detoxification enzymes in modulating malaria transmission is discussed.

## Results and Discussion

### Microarray

Tissues for microarray analyses were collected at two critical time points of the *Plasmodium *cycle in the mosquito host: 1 day following the blood meal, during which parasites invade the midgut epithelium, and 11 days after the blood meal when sporozoites are starting to be released to the hemolymph, as demonstrated by detection of parasite's DNA in the hemolymph (data not shown). The mosquitoes were fed on mice that were either infected with the parasite or uninfected. The success of infection was indirectly confirmed by randomly selecting up to 19-44 mosquitoes that were screened for the presence of oocysts (see Table S1 in Additional file 1). Most of the mosquitoes were found to be positive (70.5% to 84%) and hence it can be assumed that the tissues used in the gene expression studies were infected too.

The microarray experiment was developed to answer the following questions, regarding midgut and fat body tissues:

1. which genes respond to *Plasmodium *midgut epithelium invasion (1 day post blood meal)

2. which genes respond to the release of sporozoites into the hemolymph (11 days post blood meal), and

3. which genes respond differently between the two events (interaction term).

In the microarray analysis 146 loci were differentially expressed in at least one of the comparisons made. The results for all comparisons are given in Table S2 (Additional file 2). The microarray results were validated by comparing the mean values for the expression data (log_2 _ratio) for genes from three independent replicates obtained by microarray analysis with the corresponding mean expression values obtained with the multiplex quantitative RT-PCR. The Pearson correlation coefficient (*P *= 0.884 for midgut, *P *= 0.85 for fat body) demonstrates a high degree of correlation between the two methods (see Figure S1 in Additional file 3).

### Genes differentially expressed in infected *versus *uninfected mosquitoes at day 1 post blood meal

**Figure 1:**
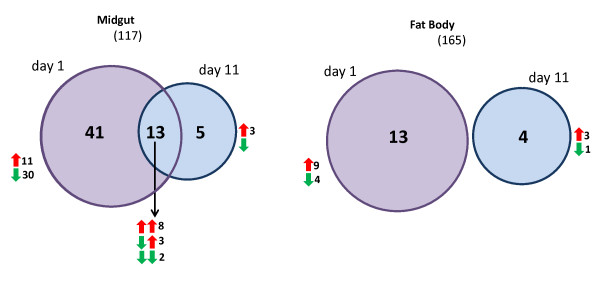
**Differential expression of detoxification genes in the midgut and fat body at day 1 and day 11 post feeding with a *P. berghei *infected or an uninfected blood meal**. The most dramatic change occurs in the midgut during sporozoite invasion (day 1 post blood feed) with 33 loci being down-regulated. While genes were predominantly down-regulated in the midgut the majority of differentially expressed genes in the fat body are up-regulated during midgut infection and sporozoite release. Numbers in brackets indicate the number of loci that were not differentially expressed at the significance cut-off level of *alpha *= 0.001.

At day 1 post blood meal more changes were observed in the midgut as compared to the fat body. While in the midgut 54 genes were differentially expressed, only 13 were different in the fat body (Figure 1, Table 1). In the midgut, 22 CYPs were differentially expressed with the majority (17) being down-regulated. In the fat body, five out of the six CYPs differentially expressed in response to *Plasmodium *infection were up-regulated. The vast majority of these differentially expressed CYPs belong to families primarily associated with detoxification roles (e.g. *CYP4*, *CYP6 *and *CYP9*) rather than families implicated in hormone biosynthetic pathways [11]. Similarly in the GST family the two classes primarily associated with xenobiotic detoxification, Delta and Epsilon [4], were generally repressed in response to parasite infection with the notable exception of *GSTD5 *which was strongly up-regulated (> 8.5 ×) in infected *vs*. uninfected midguts.

**Table 1:** Genes differentially expressed (*p *< 0.001) between infected and uninfected mosquitoes on day 1 after infection

Gene description	Probe name	1 day			
		Midgut fold	*P-value*	Fat Body fold Fold	*P-value*
**ABC transporter**	ABCC10	2.38	0		
	ABCC11	3.94	0		
	ABCC12	1.77	0		
**Actin**	Actin5C	1.44	0.0008		
**Cytochrome P450 monooxygenase**	CYP12F2	-1.93	0	2.19	0
	CYP12F4	-2.12	0		
	CYP304B1	-1.96	0		
	CYP325H1	-1.86	0.0003		
	CYP4AR1	-1.96	0.0004		
	CYP4D15	-2.70	0		
	CYP4G17			-1.26	0.00095
	CYP4H15	-1.79	0		
	CYP4H17	-2.79	0		
	CYP4H25	-2.06	0		
	CYP6AA1	-1.82	0		
	CYP6AA2	-1.93	0		
	CYP6AH1	-2.44	0		
	CYP6M1	1.60	0		
	CYP6M2	4.23	0	2.73	0
	CYP6M3	1.62	0	2.10	0
	CYP6M4	-1.29	0		
	CYP6P1	-1.38	0.0004		
	CYP6Y1			1.61	0
	CYP6Y2	1.73	0		
	CYP6Z2	-2.80	0		
	CYP9J3	-1.83	0		
	CYP9L1	-1.46	0		
	CYP9M1	1.52	0.0004	1.43	0
**Esterase**	COEAE6G	-1.52	0.00099		
	COEunkn			2.19	0.0003
**Glutathione peroxidase**	GPX2B			1.55	0
**Glutaredoxin**	GRX1	1.53	0		
**Glutathione S-transferase**	GSTD1_5	-1.56	0		
	GSTD2	-1.67	0		
	GSTD3	-1.55	0	2.17	0
	GSTD5	8.62	0.0006		
	GSTD6	-1.65	0		
	GSTD11	1.48	0		
	GSTD12	-1.49	0		
	GSTE2	-1.57	0.0001		
	GSTE3	-1.51	0		
	GSTE7	-1.84	0		
	GSTE8	1.57	0		
	GSTO1	2.90	0		
	GSTMS1	-1.46	0		
	GSTMS3	-1.36	0		
	GSTS1_2	2.08	0		
	GSTT2	-1.25	0.0002		
	GSTU2	1.91	0		
	GSTZ1			-1.42	0
**Midgut maltase-like protein**	AGM1	-1.59	0	-1.59	0
**NADPH P450 reductase**	NADPH_P450_red			-1.53	0.0002
**Nitrilase**	NIT8537			2.54	0
**Ribosomal protein**	RPL19	-1.37	0		
	RPS26	-1.53	0		
**Salivary gland protein**	GSG8	-1.43	0.0002		
**Superoxide dismutase**	SOD2	-1.98	0		
**Thioredoxin peroxidase**	TPX3	-1.47	0		
	TPX4	1.26	0.0004		
**Tubulin**	TubulinA	1.85	0		
	TubulinB	8.76	0		

In both *A. gambiae *and *A. stephensi*, *Plasmodium *parasite invasion induces an increase of nitric oxide synthase (NOS) expression and in turn an increase in nitric oxide (NO) and NO metabolites [12-15]. NO has been shown to down regulate CYP gene expression in other organisms [16]. We hypothesize therefore that the observed down-regulation of CYPs in the midgut may also be linked to increased levels of NO.

**Figure 2:**
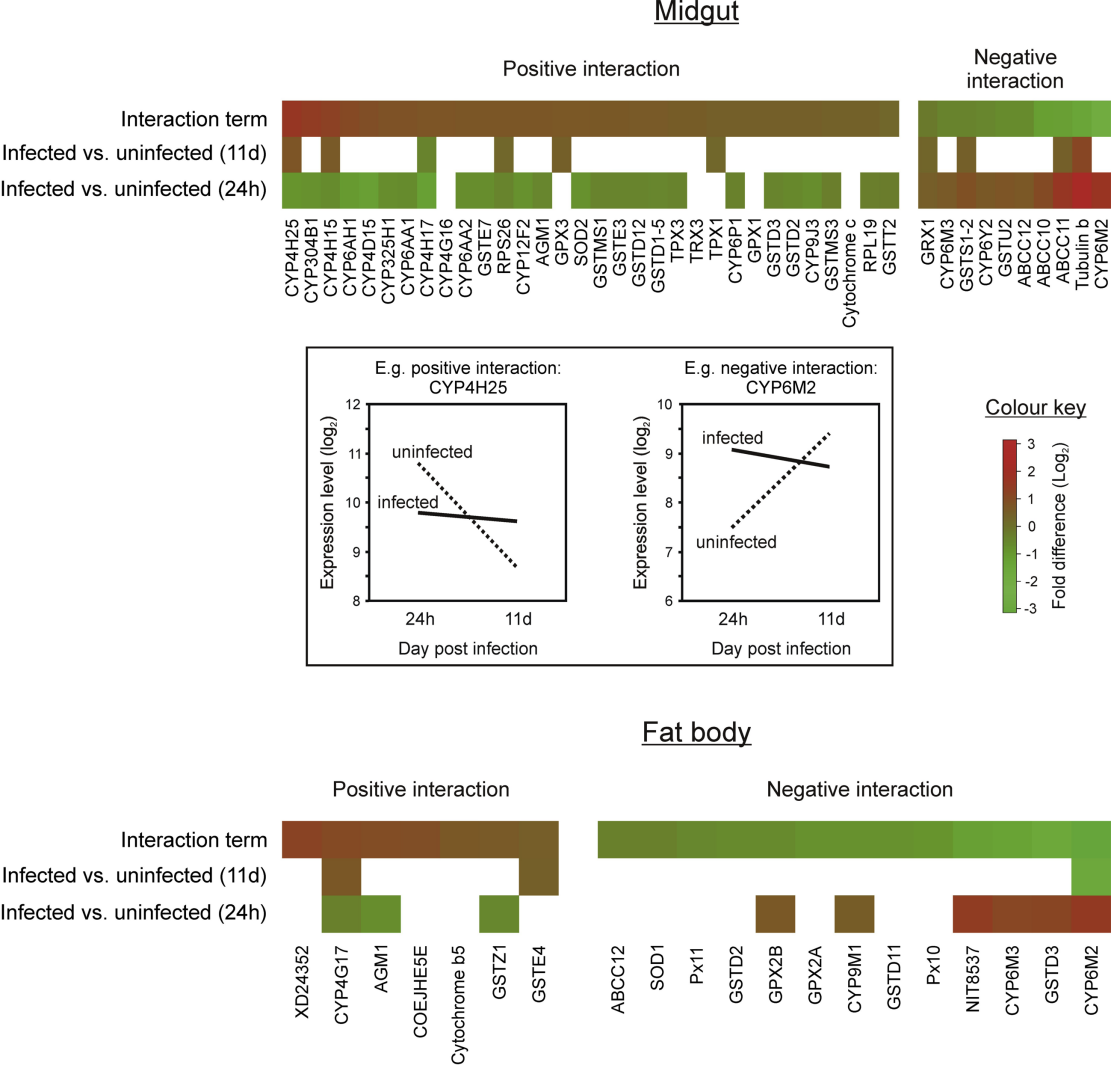
**Heat diagrams showing genes that responded differently between the event of *Plasmodium *invasion into the midgut epithelium (day 1 post feeding) and the release of sporozoites into the hemolymph (day 11 post feeding)**. The loci are plotted in the top rows and arranged from most positive interaction on the left (red) to most negative interaction (green). Inset: Examples for a positive and a negative interaction term observed in the midgut. Only loci that showed a significant interaction term (*p *< 0.001) are plotted.

Of the up-regulated CYPs, *CYP6M2 *showed the greatest response to infection (Figure 2). This gene has already been reported to be over-expressed in response to *P. berghei *infection [10] and implicated in resistance to pyrethroid insecticides [17,18]. One possible explanation for this up-regulation is a response to an endogenous mediator increased upon the infection process. As an example, prostaglandins have been shown to induce expression of CYPs in human liver cells [19].

Superoxide dismutases constitute part of the first line of defence against ROS and reactive nitrogen oxide species (RNOS) [1]. However, *SOD2 *was down-regulated 1 day post infection suggesting that down-regulation of oxidative stress response genes could be part of the defence response triggered by parasite invasion. A similar mechanism has been described for other oxidative stress response genes such as catalase in response to *Plasmodium *invasion [3].

Cytoskeleton reorganization and up-regulation of genes related to folding and movement of microtubules suggest that cytoskeleton dynamics and remodelling function as key elements of *Plasmodium *invasion of the *Anopheles *midgut [2]. This epithelium rearrangement is a robust molecular response to ookinetes penetration. In a whole genome microarray study seven tubulins were differentially up-regulated during the invasion period [2]. Here too, three cytoskeletal genes represented on the Detox array, *tubulin B*, *tubulin A *and *actin*, were up-regulated at day 1 post infection (1.85, 8.76 and 1.44 fold, respectively). In mammals, microtubule disruption leads to down-regulation of several CYPs [20] and perhaps similar responses also lead to down-regulation of CYPs during parasite invasion.

### Genes differentially expressed in infected *versus *uninfected mosquitoes 11 days post blood meal

At this time point, when sporozoites are released from oocysts to the hemocoel a less pronounced effect on the transcript levels of detoxification genes was observed as compared to midgut invasion (Table 1). Two of the genes up-regulated on day 11, *CYP4H25 *and *CYP4H15*, were down-regulated at day 1 (Table 1 and 2), suggesting that their suppression is linked to the invasion of the midgut epithelium by the parasite, while their up-regulation on day 11 may be associated with subtle changes in midgut structure as sporozoites are leaving oocysts. *GST01 *was up-regulated at both time points (Table 1 and 2) which indicates that this enzyme is directly involved in the response to parasites at both stages. Although at this stage the fat body would have had direct contact with parasites or at least molecules released by parasites during midgut egress, the transcriptional response in the fat body was more pronounced at day 1 than on day 11 post infection. *CYP6M2 *was down-regulated on day 11 but was up-regulated at day 1 (Table 1 and 2), indicating that this CYP responds to particular events of the parasites life cycle.

**Table 2:** Genes differentially expressed (*p *< 0.001) between infected and uninfected mosquitoes on day 11 after infection

Gene description	Probe name	11 days			
		Midgut fold	*P-value*	Fat Body fold	*P-value*
**ABC transporter**	ABCC11	1.47	0.0009		
**Cytochrome P450 monooxygenase**	CYP4G17			1.58	0
	CYP4H15	1.65	0		
	CYP4H17	-1.44	0.0002		
	CYP4H19	-1.65	0.0001		
	CYP4H25	1.89	0.0004		
	CYP6M2			-2.91	0
	CYP6Z2	-1.97	0		
**Glutathione peroxidase**	GPX3	1.49	0		
**Glutaredoxin**	GRX1	1.28	0		
**Glutathione S-transferase**	GSTD10	-1.65	0.0008		
	GSTD11	1.70	0		
	GSTE4			1.35	0.0003
	GSTO1	2.22	0		
	GSTS1_2	1.43	0		
**Ribosomal protein**	RPS26	1.23	0		
**Thioredoxin peroxidase**	TPX1	1.27	0.0004		
	TPX2	1.34	0		
	TPX4	1.55	0	1.65	0.0001
**Tubulin**	TubulinA	1.55	0		
	TubulinB	2.61	0		

### Genes that show a different response between *Plasmodium *midgut epithelium invasion and release of sporozoites into the hemolymph

The interaction term between the two time points was investigated to compare responses to *Plasmodium *invasion of the midgut epithelium (day 1) and to the release of sporozoites into the hemolymph (11 days). Heat diagrams with the genes that presented significant positive (increased relative expression from day 1 to day 11) and negative interaction (decreased relative expression from day 1 to day 11) in midgut and fat body are shown in Figure 2. The number of genes under positive interaction was higher in the midgut while the opposite was seen in the fat body, reflecting the active site of infection.

ABC transporters from family c showed a strong negative interaction in the midgut and to a lesser extent in the fat body, implying that these cytoplasmic membrane transporters are important for infection control probably by transporting glutathione conjugates or lipid-derived eicosanoids that are known to be involved in insect response to infection [21].

The interaction analysis confirmed that there is a considerable difference between the gene expression levels between day 1 and day 11 in response to *Plasmodium *infection. There were a high number of genes that had different levels of expression in response to the ookinetes invasion of the midgut and in response to the release of sporozoites in the hemolymph, showing that these genes have the ability of changing their expression levels according with the time of infection.

In the midgut, the majority (69%) of differentially expressed genes between day 11 and day 1 were the same both in uninfected and infected mosquitoes, as was the direction of change, indicating that these genes were responding mainly to the blood meal, as it represents a strong oxidative insult. However, this total concordance was not observed in the fat body where only 26% of genes were regulated in the same direction between infected and uninfected while 38% were regulated in opposite directions (see Table S2 in Additional file 2). The trend of expression of both tissues suggests that differences observed are due to fat body response to parasite released from the oocysts into the hemocoel.

The mosquito response to sporozoites in the hemolymph triggers effector mechanisms like melanization [1], and a burst of expression of genes encoding constituents of the immune system including the production of free radicals [12] that needs a counter detoxification reaction. After excluding genes similarly regulated in both infected and uninfected groups, fat body CYP genes were down-regulated, at day 11, as observed for the midgut at day 1. *SOD2 *was down-regulated and seems to be determinant for parasite control. *TPX4 *was up-regulated confirming its role on infection detoxification mediated by the fat body. The fat body has an important role in the detoxification and in the immune response of the mosquito on day 11 of infection when compared with day 1 post infection, which is not observed when we compare infected and uninfected mosquitoes on day 11.

## Conclusions

This study determined transcription profiles of detoxification enzymes during *Plasmodium *infection in *A. gambiae*, showing important changes in the expression of several detoxification enzymes, as well as membrane associated ABC transporters. Interestingly, genes coding for detoxification enzymes revealed a variable response, being differentially induced or repressed depending on the tissue and stage of infection.

**Figure 3:**
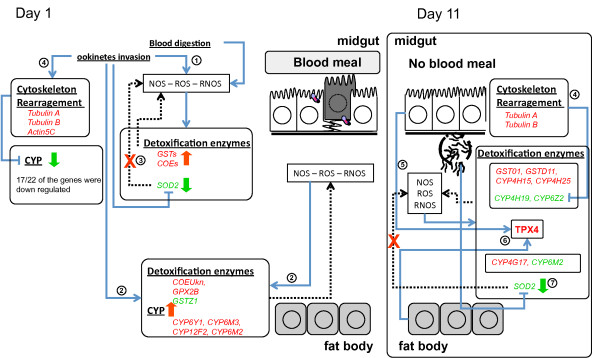
**Hypothetical scenario of *Anopheles gambiae *detoxification response to *Plasmodium berghei *infection**. Midgut and fat body genes up-regulated (red) and down-regulated (green) after infection at 2 different time points, day 1 (invasion of midgut epithelium by ookinetes) and day 11 (sporozoite egress from oocysts), and between the two events. At day 1, blood digestion and parasite invasion cause an increase in the ROS and RNOS that consequently increases the expression of detoxification enzymes (1). The parasite invasion and the ROS and RNOS also affect the fat body increasing the expression of detoxification enzymes in this tissue (2). At the same time midgut cells in response to parasite invasion suppresses the SOD expression (3) as a mechanism to eliminate parasites. Parasite invasion of midgut epithelium causes a massive cytoskeleton rearrangement that down regulates CYPs expression (4). On day 11, there is no blood digestion, but oocysts burst and sporozoites are released to the hemolymph. In the midgut the oocysts burst provokes a cytoskeleton rearrangement (4), as in day 1, that probably also down regulates CYPs expression in the midgut. While in the hemolymph sporozoites cause an increase in the ROS and RNOS that increase the detoxification enzymes expression in both midgut and fat body (5). Here, both midgut and fat body altered the expression of *TPX4 *(6), that is essential for hydrogen peroxide detoxification through the thioredoxin system. In the fat body sporozoites also provoke suppression of SOD expression (7).

Although the mechanism underlying these changes is presently unclear, this differential regulation of detoxification genes observed during *Plasmodium *infection may be due to 1) the increasing oxidative stress caused by the presence of the parasite; 2) the epithelium rearrangement involving alterations in cytoskeleton genes caused by the ookinetes invasion and the oocysts burst; or 3) a combination of both. A hypothetical scenario for the inter-relationship between infection and detoxifying molecules is depicted in Figure 3.

In contrast to the majority of differentially expressed P450s which were down-regulated in response to midgut invasion, *CYP6M2 *expression was induced in response to *P. berghei *infection. This CYP is involved in resistance to pyrethroid insecticides [17,18]. This observation together with regulation of other genes, such as membrane ABC transporters involved in xenobiotic elimination, lead us to speculate that there might be an association between the response to *Plasmodium *infection and insecticide resistance, enhancing the importance of further studying their interaction.

## Methods

### Mosquitoes

*A. gambiae *s.s. (molecular M form) of the Yaoundé strain mosquitoes were reared at 26°C and 75% relative humidity on a 12/12 hours light/dark cycle. Adult mosquitoes were maintained on 10% glucose solution until blood feeding.

### *P. berghei *infection of mosquitoes

Female CD1 mice were intraperitoneally inoculated with 10^7 ^*P. berghei *ANKA parasitised red blood cells. The levels of parasitaemia were measured from blood samples of the mouse tail using Giemsa-stained blood films. When the parasitaemia reached 10-20% and exflagellation was observed, mice were used to infect mosquitoes. Female mosquitoes were allowed to feed directly on naïve (control) and *P. berghei *infected mice up to one hour, with regular monitoring to certify mice were anesthetised. Fully engorged mosquitoes were kept at 19-21°C and 80% relative humidity for P. berghei development. The maintenance and care of experimental animals complied with portaria n° 1005/92 from 23rd October and was approved by the Divisão Geral de Veterinaria, Portugal.

### Tissue collection

Mosquito midguts and abdominal walls containing fat body tissues were collected from pools of 40 sibling mosquitoes at day 1 and on day 11 after the blood meal. This procedure was repeated to obtain 3 independent replicates. Tissues were dissected from mosquitoes submerged in ice-cold phosphate-buffered saline (PBS) that was prepared with DEPC-treated water and transferred to ice-cold RNAlater (Ambion). After incubation at 4°C over night any excess RNAlater was removed and samples were stored at -20°C until RNA extraction. On day 11 post infection mosquito midguts were also collected to determine infection rate (number of infected mosquitoes over total number of mosquitoes observed).

### Microarray analysis

Protocols for RNA extraction, amplification and labelling with fluorescent dyes are described in [22]. Fluorescent Cy3- and Cy5-labelled targets were hybridised to the latest version of the *A. gambiae detox chip *[23] (ArrayExpress accession AMEXP-863). The features on this version of the *detox chip *probe for 103 cytochrome P450s, 31 esterases, 35 glutathione S-transferases and 85 additional loci coding for enzymes such as peroxidases, reductases, superoxide dismutases, ATP-binding cassette transporters, tissue specific genes and housekeeping genes.

Two separate microarray experiments were conducted; one for the RNA pools obtained from midguts and another one for RNA extracted from fat body tissues. Each experiment followed a 2 × 2 factorial design in which the first factor was *time *and the second one was *infection status*. Each factor was measured at two levels; at one and eleven days post blood meal and from female mosquitoes that were either fed with *Plasmodium*-infected or uninfected blood. Factors and levels were combined constituting a total of four measurements. Each combination was repeated three times with tissues from 40 individuals pooled for RNA isolation per replicate (see Figure S2 in Additional file 4).

After scanning of raw signal intensities and visual spot inspection in GenePix Pro 5.1 software (Axon Instruments) data were exported to *limma *(version 2.9). *Limma*, part of the Bioconductor project [24], is a bioinformatics package for the analysis of linear models in microarray experiments [25] implemented in R http://www.r-project.org. Here, median spot and background intensities from the red (Cy5) and green (Cy3) channels were analysed. Any spot with a saturated signal in either the green or the red channel was excluded from the statistical analysis. For each spot, background intensities were first subtracted from the foreground intensities. To generate positive corrected intensities any intensity that was less than 0.5 after background subtraction was reset to 0.5. Background-corrected intensities from each spot were then transformed to intensity log-fold changes, *M *= log_2_(*red*)-log_2_(*green*), and their geometrical means, A = [log_2_(*red*)+log_2_(*green*)]/2. Within each array, *M*-values for each spot were subsequently normalized as a function of *A *using the loess scatter plot smoothing function implemented in *limma*. In the normalization step the calibration spots on the *detox chip *were included too. The *detox chip *contains 40 calibration spots representing a 1:1 dilution series over a concentration gradient from 1 pg to 30 ng per 2 μl of added mRNA spike-in mix).

For the statistical analysis of the microarray experiments *limma *employs a linear model approach whereby linear models are fitted to the normalised data for each locus probed by the array [25,26]. Because each unique probe is spotted four times onto the *detox chip *we took advantage of the pooled correlation method implemented in *limma *to make full use of the replicate spots [27]. Contrasts, linear combinations of the coefficients, were then tested for significance. The contrasts tested between factor levels (*time *and *infection status*) and the interaction term (*time *× *infection status*) are given in Figure S2 (Additional file 4). To assess differential expression *limma *uses an empirical Bayes method to moderate the standard errors of the estimated log-fold changes [26]. This approach results in more stable inference and improved power, especially for experiments with small numbers of arrays [28]. *P*-values obtained from the *t*-tests (with the moderated *t*-statistic) were adjusted for multiple testing adopting the approach of Benjamini and Hochberg [25,29]. In order to define a set of differentially expressed genes only hits with an adjusted *p*-value below the level of significance, *α *= 0.001, were considered.

All microarray data have been deposited in ArrayExpress (ArrayExpress accession E-MTAB-195).

### Quantitative RT-PCR

To validate microarray data a subset of 20 differentially expressed genes (see Table S2 in Addditional file 2) were chosen and their expression levels measured by multiplexed quantitative RT-PCR. The same RNA pools used in the microarray experiment served as target RNA in the PCR. The Beckman Coulter GeXP system was used to quantify the expression of these genes and the ribosomal protein RPS7-encoding gene [VectorBase: AGAP010592] was used for normalisation as described in [22]. PCR primer sequences are given in Table S3 (see Additional file 5).

## Authors' contributions

RF and PM performed the experiments; RF, PM and HS analyzed and interpreted the data; RF, PM, HR and HS wrote the paper. RF, PM, HS and VR conceived and designed the experiments. All authors read and approved the final manuscript.

## Supplementary Material

Additional file 1 **Table S1**. Infection rate and oocyst load of *A. gambiae *infected with *P. berghei *used for the microarray experiments. Click here for file

Additional file 2 **Table S2**. List of all the genes differentially expressed (p < 0.001) represented on the *Detox *chip including fold change in expression and *p*-values. Click here for file

Additional file 3 **Figure S1**. Validation of the DNA microarray analysis using quantitative RT-PCR. The mean expression values for midgut genes (A) and fat body genes (B) obtained by microarray analysis were plotted against the corresponding mean expression values obtained with quantitative RT-PCR. A high level of consistency between the two datasets was demonstrated by the Pearson correlation coefficient (P = 0.884) for midgut and (P = 0.85) for fat body and best-fit linear-regression analysis (R^2 ^= 0.7814) for midgut and (R^2 ^= 0.7228) for fat body. Click here for file

Additional file 4 **Figure S2**. Design of the microarray experiments. The experiments for midgut and fat body tissues followed the same layout. The boxes of the graphs represent RNA extracted from pools of 40 individuals and the arrows the microarrays to which labelled target RNA was co-hybridized. The tails of the arrows represent the samples that were labelled with a green (Cy3) and the heads those samples that were labelled with a red (Cy5) fluorescent dye. For the design matrix in *limma*, the samples from uninfected tissues collected 1 day post infection were set as the reference pool (shaded boxes). After fitting linear models the contrasts shown below the diagram were constructed for hypothesis testing of specific comparisons between RNA pools. For each of the three biological blocks (replicates 1 to 3) and factor combination a separate coefficient was included in the design matrix. The contrasts were extracted by taking the average of the three comparisons. Click here for file

Additional file 5 **Table S3**. Sequences of oligonucleotide primers used in quantitative RT-PCR validation experiments. Click here for file
