# Supplementary material for: Plasmodium infection alters Anopheles gambiae detoxification gene expression
Source: BMC Genomics. 2010 May 19;11:312. doi: 10.1186/1471-2164-11-312 (PMC2885368; doi:10.1186/1471-2164-11-312)
Supplement: Additional file 1 — Table S1. Infection rate and oocyst load of A. gambiae infected with P. berghei used for the microarray experiments. [file 1471-2164-11-312-S1.PDF]

	Experiment 1	Experiment2	Experiment 3
N	44	24	19
Infection Rate	70.5	75	84
Median number of oocysts by midgut	60	70.33	50.75
