# Supplementary material for: Plasmodium infection alters Anopheles gambiae detoxification gene expression
Source: BMC Genomics. 2010 May 19;11:312. doi: 10.1186/1471-2164-11-312 (PMC2885368; doi:10.1186/1471-2164-11-312)
Supplement: Additional file 3 — Figure S1. Validation of the DNA microarray analysis using quantitative RT-PCR. The mean expression values for midgut genes (A) and fat body genes (B) obtained by microarray analysis were plotted against the corresponding mean expression values obtained with quantitative RT-PCR. A high level of consistency between the two datasets was demonstrated by the Pearson correlation coefficient (P = 0.884) for midgut and (P = 0.85) for fat body and best-fit linear-regression analysis (R2 = 0.7814) for midgut and (R2 = 0.7228) for fat body. [file 1471-2164-11-312-S3.PDF]

Gene	VectorBase Gene ID	Left Sequence w/Universals (5'-3')	Right Sequence w/Universals (5'-3')
**CYP6Y1**	AGAP008208	AGGTGACACTATAGAATAGAAGCAGACGCTCCAGAAGT	GTACGACTCACTATAGGGAATGTTTGCGCAGTGTCTCAC
**CYP4G17**	AGAP000877	AGGTGACACTATAGAATACGATGGCTGTCATGAAAATG	GTACGACTCACTATAGGGACTCCTGTTCCTTCTTGACGC
**NIT8537**	AGAP003515	AGGTGACACTATAGAATAGCGTACGGCTATTCGTTGAT	GTACGACTCACTATAGGGACTTAATCTCGCCGCACATCT
**CYP6P3**	AGAP002865	AGGTGACACTATAGAATAAGCTAATTAACGCGGTGCTG	GTACGACTCACTATAGGGAAAGTGTGGATTCGGAGCGTA
**ABCC11**	AGAP008436	AGGTGACACTATAGAATATCATCTACCGGGACTTTTCG	GTACGACTCACTATAGGGATCCCAATGAAGCTGGATTTC
**CYP304B1**	AGAP003066	AGGTGACACTATAGAATAGTTCAGCTGCTTTGCCAAC	GTACGACTCACTATAGGGAGCTGAGAATCGTGCCGTAGT
**COEBE3C**	AGAP005372	AGGTGACACTATAGAATAAGCTCATGCATCCCTTCACT	GTACGACTCACTATAGGGAGACGCTGGGGAATATTAGCA
**GSTD11**	AGAP004378	AGGTGACACTATAGAATAGCTGACGAGCATCACTACCA	GTACGACTCACTATAGGGAGTTGATCGGGTTGAACGAGT
**CYP6M3**	AGAP008213	AGGTGACACTATAGAATATCAAGTACCGGGTGGAGAAC	GTACGACTCACTATAGGGACAGCGTGGAAGATGTCTCAA
**Tubulin B**	AGAP010510	AGGTGACACTATAGAATAGCTACCTAACAGTCGCTGCC	GTACGACTCACTATAGGGATTACCAATGAACGTGGACGA
**TPX4**	AGAP011824	AGGTGACACTATAGAATAAAAAGCGCAATGTGAAGGTC	GTACGACTCACTATAGGGACTCCACGTTGTCCTTGTCCT
**CYP6M2**	AGAP008212	AGGTGACACTATAGAATATTCGTCGACTCTCCTCACCT	GTACGACTCACTATAGGGAGAAATGTACCGGGACTGGTG
**GSTS1-2**	GenBank: AF513639	AGGTGACACTATAGAATACGGTGAACGATTTCCGTCTA	GTACGACTCACTATAGGGAATAGTCCAAAATGGCGGTGA
**AGM1**	AGAP012401	AGGTGACACTATAGAATAGTGTATCCGGACGAGGAGAA	GTACGACTCACTATAGGGACCGAAACTTCATTGCCAAAT
**GSTE2**	AGAP009194	AGGTGACACTATAGAATACTGCGAAAATGTCCAACCTT	GTACGACTCACTATAGGGATTTGCCATACTTCGTCACCA
**CYP4G16**	AGAP001076	AGGTGACACTATAGAATAGCCTTAGACCTTGTTGGCAG	GTACGACTCACTATAGGGAGCAATCAGTTTGCGATGTTG
**CYP9L3**	AGAP012293	AGGTGACACTATAGAATAATCGGAGACACTGCGAAAGT	GTACGACTCACTATAGGGAATGCAATTGCGTGGTCCTAT
**CYP9J5**	AGAP012296	AGGTGACACTATAGAATAGAAGGATGTGTTTACGCGGT	GTACGACTCACTATAGGGAAACCATATCGGGTCGAACAA
**XD24352**	AGAP007918	AGGTGACACTATAGAATAAAATGATTCAGGTTGCTGCC	GTACGACTCACTATAGGGAATTGGTGCCAAAATCGTAGC
**CYP12F2**	AGAP008021	AGGTGACACTATAGAATAAAATTCCAAAGGGAACGGAC	GTACGACTCACTATAGGGAGGATTGGCAGGAATGTTGAT
**RPS7**	AGAP010592	AGGTGACACTATAGAATACATTTCGTTGTGAACCCAAA	GTACGACTCACTATAGGGAAGTTCATCTCCAGCTCCAGG
