# Supplementary figures and images for: Plasmodium infection alters Anopheles gambiae detoxification gene expression
Source: BMC Genomics. 2010 May 19;11:312. doi: 10.1186/1471-2164-11-312 (PMC2885368; doi:10.1186/1471-2164-11-312)

A)

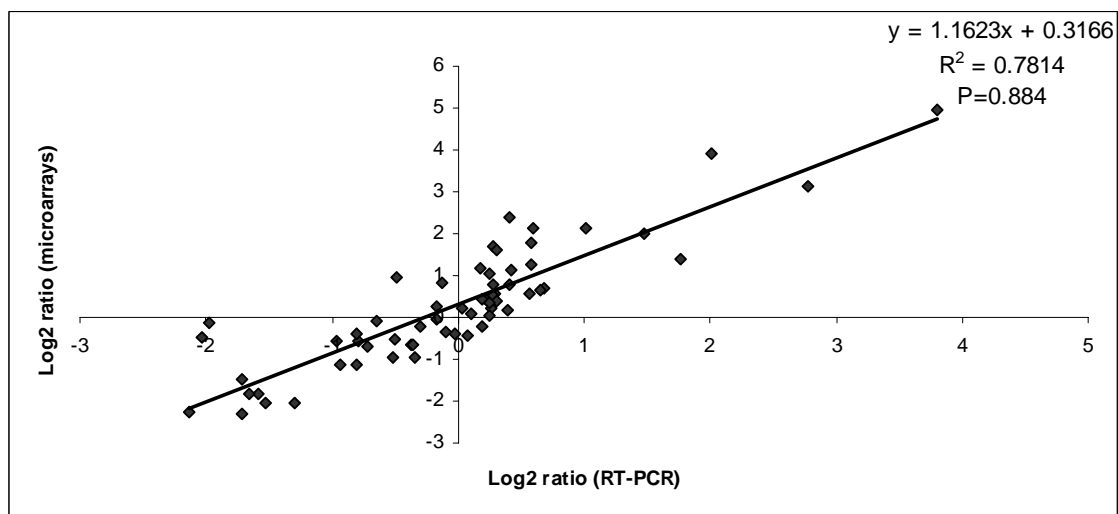

B)

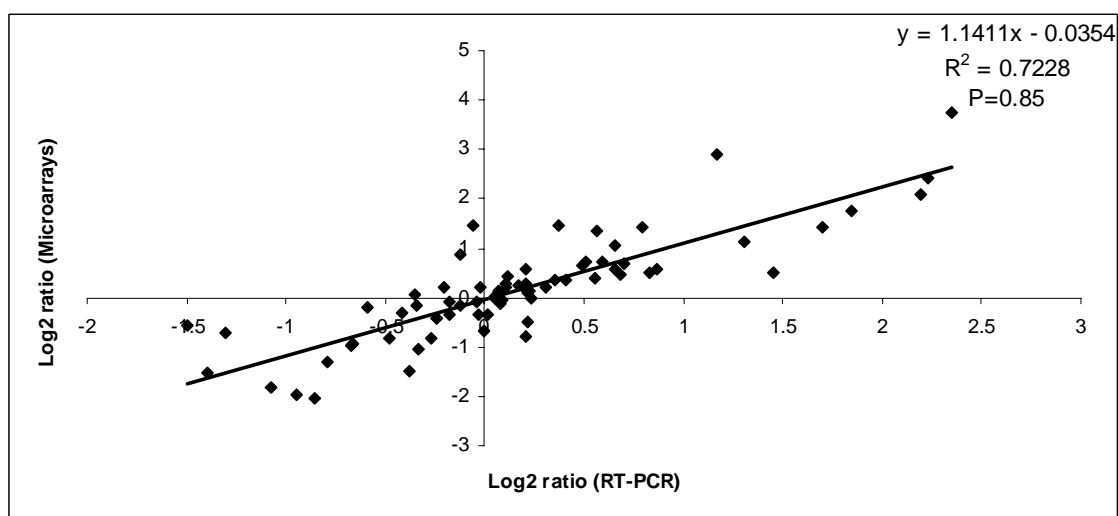

Supplement: Additional file 4 — Figure S2. Design of the microarray experiments. The experiments for midgut and fat body tissues followed the same layout. The boxes of the graphs represent RNA extracted from pools of 40 individuals and the arrows the microarrays to which labelled target RNA was co-hybridized. The tails of the arrows represent the samples that were labelled with a green (Cy3) and the heads those samples that were labelled with a red (Cy5) fluorescent dye. For the design matrix in limma, the samples from uninfected tissues collected 1 day post infection were set as the reference pool (shaded boxes). After fitting linear models the contrasts shown below the diagram were constructed for hypothesis testing of specific comparisons between RNA pools. For each of the three biological blocks (replicates 1 to 3) and factor combination a separate coefficient was included in the design matrix. The contrasts were extracted by taking the average of the three comparisons. [file 1471-2164-11-312-S4.PDF]
