# Supplementary material for: Plasmodium infection alters Anopheles gambiae detoxification gene expression
Source: BMC Genomics. 2010 May 19;11:312. doi: 10.1186/1471-2164-11-312 (PMC2885368; doi:10.1186/1471-2164-11-312)
Supplement: Additional file 5 — Table S3. Sequences of oligonucleotide primers used in quantitative RT-PCR validation experiments. [file 1471-2164-11-312-S5.PDF]

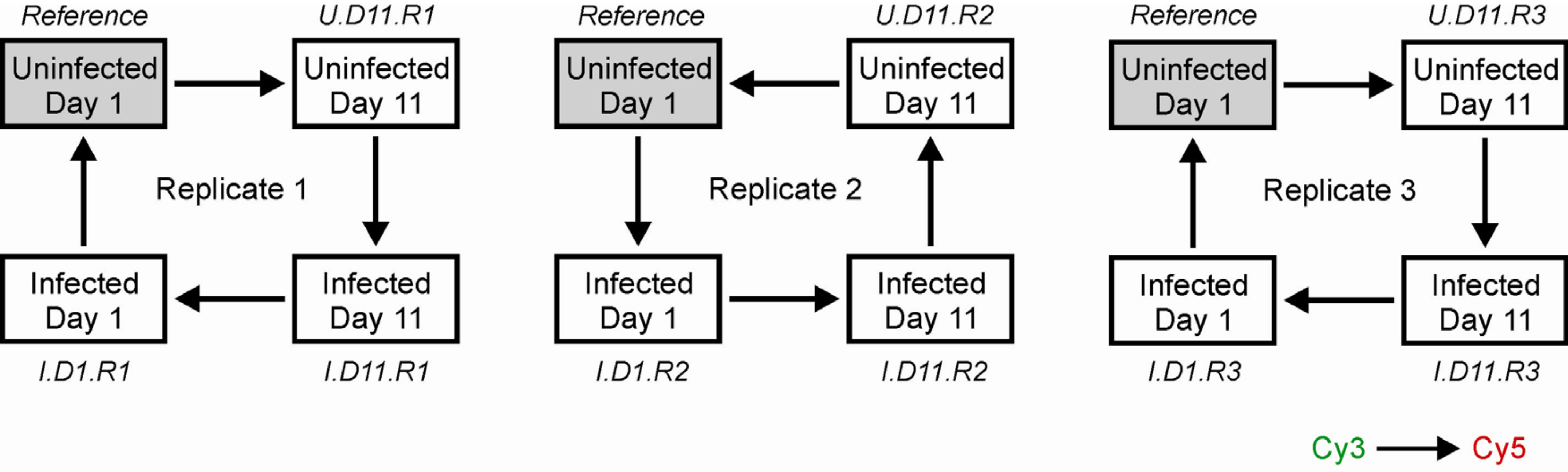

**Term**	**Contrast**
Infected vs. uninfected (1 day)	$\frac{I.D1.R1 + I.D1.R2 + I.D1.R3}{3}$
Infected vs. uninfected (11 days)	$\frac{I.D11.R1 - U.D11.R1 + I.D11.R2 - U.D11.R2 + I.D11.R3 - U.D11.R3}{3}$
Interaction	$\frac{(I.D11.R1 - I.D1.R1 + I.D11.R2 - I.D1.2 + I.D11.R3 - I.D1.3) - (U.D11.R1 + U.D11.R2 + U.D11.R3)}{3}$
